# Supplementary material for: Facilitators and Barriers to Physical Activity and Sport Participation Experienced by Aboriginal and Torres Strait Islander Adults: A Mixed Method Review
Source: Int J Environ Res Public Health. 2021 Sep 20;18(18):9893. doi: 10.3390/ijerph18189893 (PMC8468807; doi:10.3390/ijerph18189893)
Supplement: Supplementary file 1 [file ijerph-18-09893-s001.zip › ijerph-1366994-supplementary.pdf]

Table S1. Aboriginal and Torres Strait Islander Quality Appraisal Tool.

First Author/Year	1. Did the research respond to a need or priority determined by the community?	2. Was community consultation and engagement appropriately inclusive?	3. Did the research have Aboriginal and Torres Strait Islander research leadership?	4. Did the research have Aboriginal and Torres Strait Islander governance?	5. Were local community protocols respected and followed?	6. Did the researchers negotiate agreements in regards to rights of access to Aboriginal and Torres Strait Islander peoples existing intellectual and cultural property?	7. Did the researchers negotiate agreements to protect Aboriginal and Torres Strait Islander peoples ownership of intellectual and cultural property created through the research?	8. Did Aboriginal and Torres Strait Islander peoples and communities have control over the collection and management of research materials?	9. Was the research guided by an Indigenous research paradigm?	10. Does the research take a strengths-based approach, acknowledging and moving beyond practices that have harmed Aboriginal and Torres Strait peoples in the past?	11. Did the researchers plan and translate the findings into sustainable changes in policy and/or practice?	12. Did the research benefit the participants and Aboriginal and Torres Strait Islander communities?	13. Did the research demonstrate capacity strengthening for Aboriginal and Torres Strait Islander individuals?	14. Did everyone involved in the research have opportunities to learn from each other?
Andrews 2013 [34]	Y	P	P	P	P	U	U	U	Y	Y	P	Y	Y	Y
Canuto 2013 [39]	Y	Y	Y	Y	U	N	N	U	P	Y	U	Y	P	Y
Caperchione 2009 [40]	U	U	U	U	U	N	N	U	U	P	P	U	U	U
Carr 2019 [35]	Y	Y	Y	Y	Y	N	N	Y	Y	Y	Y	Y	U	Y
Cavanagh 2015 [54]	U	U	U	U	P	U	U	P	N	P	P	U	U	P
Davey 2014 [53]	Y	U	P	P	U	Y	U	U	U	P	U	Y	Y	U
David 2018 [18]	Y	U	N	U	Y	U	U	Y	N	Y	Y	U	Y	Y
Hunt 2008 [42]	U	Y	Y	N	U	U	U	U	N	N	U	U	Y	U
Lin 2012 [37]	Y	Y	U	Y	U	N	N	U	U	Y	P	Y	Y	Y
Macdonald 2012 [43]	U	U	U	U	U	U	U	P	P	Y	U	U	Y	U
Macniven 2018 [36]	Y	Y	Y	U	Y	U	U	Y	Y	Y	Y	Y	U	Y
Macniven 2020 [49]	U	P	U	U	Y	U	U	U	Y	Y	P	U	P	P
Maxwell 2019 [50]	U	U	U	U	U	U	U	P	Y	Y	U	U	U	U
Mellor 2016 [41]	U	U	U	U	U	U	U	U	N	N	U	U	Y	U

Nalatu 2019 [32]	Y	Y	U	Y	Y	U	U	U	U	Y	P	Y	U	Y
Nelson 2016 [44]	U	U	U	Y	U	N	N	Y	U	P	U	P	N	U
Parmenter 2020 [45]	P	Y	U	N	Y	N	N	N	Y	Y	Y	Y	U	U
Peloquin 2017 [46]	U	U	U	U	U	U	U	U	N	P	P	U	U	U
Seear 2019 [52]	U	U	N	P	P	U	U	P	Y	P	P	U	Y	U
Stronach 2016 [31]	U	P	Y	U	U	N	N	P	Y	Y	U	U	N	U
Stronach, Adair, Maxwell 2019 [30]	U	U	N	U	U	U	U	N	Y	Y	U	U	N	U
Stronach, Maxwell, Pearce 2019 [29]	U	U	Y	U	U	U	U	U	Y	Y	P	U	P	P
Sushames 2017 [47]	U	U	N	N	U	U	U	U	N	N	U	U	N	U
Thompson 2013 [51]	P	Y	U	U	U	U	U	U	N	Y	U	U	Y	U
Thorpe 2014 [38]	U	Y	Y	Y	Y	U	U	Y	Y	P	P	U	Y	P
Walker 2020 [48]	N	P	Y	P	U	N	N	U	N	Y	P	U	N	U
Young 2018 [33]	N	U	U	N	U	U	U	U	U	P	P	P	P	U

Key: Y = yes; N = no; U = unclear; P = partially.

Table S2. Mixed Methods Appraisal Tool.

**First Author/Year**	Screening 1. Are there clear research questions?	Screening 2. Do the collected data allow to address these research questions?	1.1. Is the **qualitative** approach appropriate to answer the research question?	1.2. Are the qualitative data collection methods adequate to address the research question?	1.3. Are the findings adequately derived from the data?	1.4. Is the interpretation of results sufficiently substantiated by data?	1.5. Is there coherence between qualitative data sources, collection, analysis and interpretation?	5.1. Is there an adequate rationale for using a **mixed methods** design to address the research question?	5.2. Are the different components of the study effectively integrated to answer the research question?	5.3. Are the outputs of the integration of qualitative and quantitative components adequately interpreted?	5.4. Are divergences and inconsistencies between quantitative and qualitative results adequately addressed?	5.5. Do the different components of the study adhere to the quality criteria of each tradition of the methods involved?
Andrews 2013 [34]	Y	Y	Y	Y	Y	Y	Y					
Canuto 2013 [39]	Y	Y						Y	Y	Y	Y	Y
Carr 2019 [35]	Y	Y	Y	Y	Y	Y	Y					
Caperchoine 2009 [40]	Y	Y	Y	Y	Y	Y	Y					
Cavanagh 2015 [54]	Y	Y	Y	Y	Y	Y	Y					
Davey 2014 [53]	Y	Y						Y	Y	Y	Y	Can't tell
David 2018 [18]	**Y**	Y						Y	Y	Can't tell	N	N
Hunt 2008 [42]	Y	Y	Y	Y	Y	Y	Y					
Lin 2012 [37]	Can't tell	Y	Y	Y	Y	Y	Y					
Macdonald 2012 [43]	Y	Y	Y	Y	Y	Y	Y					
Macniven 2018 [49]	Y	Y						Y	Y	Y	Y	Y
Macniven 2020 [36]	Y	Y						Y	N	Y	N	Y
Maxwell 2019 [50]	Y	Y	Y	Y	Y	Y	Can't tell					
Mellor 2016 [41]	Y	Y	Y	Y	Y	Y	Y					
Nalatu 2012 [32]	Y	Y	Y	Y	Y	Y	Y					
Nelson 2016 [44]	Y	Y						Y	Y	Y	Y	Can't tell
Parmenter 2020 [45]	Y	Y	Y	Y	Y	Y	Y					
Peloquin 2017 [46]	Y	Y	Y	Y	Y	Y	Y					
Seear 2019 [52]	Y	Y	Y	Y	Y	Y	Y					
Stronach 2016 [31]	Y	Y	Y	Y	Y	Y	Y					
Stronach, Adair, Maxwell 2019 [30]	Y	Y	Y	Can't tell	Can't tell	Can't tell	Can't tell					
Stronach, Maxwell, Pearce 2019 [29]	Y	Y	Y	Y	Y	Y	Y					
Sushames 2017 [47]	Y	Y	Y	Y	Y	Y	Y					
Thompson 2013 [51]	Y	Y	Y	Y	Y	Y	Y					
Thorpe 2014 [38]	Y	Y	Y	Y	Y	Y	Y					
Walker 2020 [48]	Y	Y	Y	Y	Y	Y	Y					
Young 2018 [33]	Y	Y	Y	Y	Y	Y	Y					
